# Compensatory Movements of the Midfoot Joints Influence Gait Pattern After Arthroscopic Ankle Arthrodesis

**DOI:** 10.1177/24730114251338848

**Published:** 2025-06-03

**Authors:** Annette Eidmann, Katharina Kraftborn, Matthias G. Walcher, Lukas Fraißler, Maximilian Rudert, Ioannis Stratos

**Affiliations:** 1 Julius-Maximilians University Wuerzburg, Department of Orthopaedic Surgery, Koenig-Ludwig-Haus, Wuerzburg, Germany; 2 OCW Orthopädie Chirurgie Wuerzburg, Wuerzburg, Germany; 3 Privatordination für Fuß- und Sprunggelenkchirurgie, Privatklinikum Hansa, Graz, Austria

**Keywords:** arthroscopic ankle arthrodesis, gait analysis, peak pressure, compensatory movements

## Abstract

**Background:**

Arthroscopic ankle arthrodesis (AAA) is a standard procedure for end-stage osteoarthritis of the ankle. One of the main concerns after AAA remains the development of secondary osteoarthritis in the subtalar and tarsal joints in the long term. This development is thought to be due to a compensatory increased mobility and therefore increased load on the adjacent joints. Therefore, the aim of the study was to analyze the residual motion of the tarsal joints, the load distribution under the foot, and the influence of tarsal joint motion on load distribution and gait pattern after AAA.

**Methods:**

29 patients with arthroscopic AAA were analyzed in a retrospective case-control series by pedobarographic gait analysis and fluoroscopy. The variables examined by pedobarography included peak force, peak pressure, and contact time of 10 different zones of the foot during the roll-over process, comparing the operated with the contralateral healthy foot. The range of motion (ROM) of the subtalar and medial tarsal joints in dorsiflexion/plantarflexion were assessed radiologically.

**Results:**

After AAA, peak forces of the ipsilateral foot were significantly reduced for the entire foot and especially the first metatarsal, great toe, and lesser toes during the roll-over process. Peak pressure decreased significantly under the lesser toes and increased under metatarsal 5, without significant load alterations under the mid- and hindfoot. The residual ROM of the subtalar and tarsal joints in dorsiflexion/plantarflexion was 23.5 degrees. The greater the ROM of the adjacent joints, the more the gait pattern normalized.

**Conclusion:**

Load distribution during the stance phase is influenced by AAA; the ROM of the subtalar and midfoot joints is essential in normalizing gait pattern.

**Level of Evidence:**

IV, case series.

## Introduction

Despite advances in total ankle replacement in recent years, arthrodesis of the tibiotalar joint remains a standard procedure for end-stage osteoarthritis of the ankle. In particular, minimally invasive arthroscopic ankle arthrodesis (AAA) has become increasingly popular because of its advantages in terms of complication rates, especially soft tissue complications, length of hospital stay, and blood loss.^9,12,15,16^ AAA can achieve very good functional results with a high level of patient satisfaction, particularly with regard to pain relief.^7,16,26^ Although the mobility of a large joint is sacrificed, the subjective restrictions when walking are often perceived as minor and the gait pattern is demonstrably improved by arthrodesis compared with the preoperative, arthritic joint.^4,22^ Nevertheless, the stiffening of the tibiotalar joint affects the propulsion phase of gait and can therefore lead to a changed gait pattern and limitations in certain activities.^28^ Some studies have investigated the altered gait pattern, especially focusing on spatiotemporal variables like gait speed, step length, cadence, and time and duration of the stance phase.^3,8,10,20,22,27^ However, there is still little evidence on the load on the adjacent joints, especially the tarsal joints. The mobility of the adjacent joints is thought to be essential to compensate for the reduced range of motion of the ankle joint during gait.^14^ In addition to the knee and hip joint, this primarily affects the subtalar joint and the joints of the midfoot. It is assumed that the compensatory movements of those joints cause an additional load, which favors the development of secondary osteoarthritis.^1^ Osteoarthritis of the adjacent joints is one of the main concerns after ankle arthrodesis. In the long term, almost all patients after tibiotalar arthrodesis develop radiologically detectable osteoarthritis in the subtalar joint and many also in the joints of the midfoot. There is still no clear evidence that arthrodesis of the tibiotalar joint is causal for the development of secondary osteoarthritis and that osteoarthritis is clinically relevant.^11^ Nevertheless, subsequent arthrosis is cited as one of the disadvantages compared to total ankle replacement, as secondary arthrosis of the subtalar and tarsal joints appears to be less common here.^5,21^

To investigate the effects of tibiotalar arthrodesis on the adjacent joints, we analyzed the remaining range of motion of the subtalar and the tarsal joints via fluoroscopy. To examine the load on the different areas of the foot, we used pedobarographic gait analysis. Pedobarography is an established procedure for carrying out detailed information on ground reaction forces during gait on different areas of the foot.^17^ In order to test the hypothesis that the mobility of the adjacent joints is associated with the gait pattern and the load on the neighboring joints, correlation analyses were employed on the variables of interest. Subtalar and tarsal joint compensation was defined as the increased motion in adjacent joints that occurs following loss of mobility due to ankle arthrodesis. “Better” compensation refers to a level of residual motion in these joints that restores gait parameters closer to physiological patterns seen in the nonoperated foot. Conversely, “worse” compensation would imply either insufficient mobility leading to impaired gait.

## Material and Methods

### Study Population

For this retrospective study, all patients aged between 18 and 80 years who underwent primary arthroscopic ankle arthrodesis (AAA) between February 2014 and March 2017 were selected for analysis. Revision arthrodesis, additional surgery of adjacent joints, and open surgery were excluded. Patients with bilateral arthrodesis had to be excluded from pedobarographic analysis because of the lack of a healthy foot for control. The follow-up period had to be at least 5 months postoperatively in order to ensure that the test subjects were able to safely bear full weight. The study was approved by the local ethics committee (number 20160810 01).

### Testing Protocol

All patients received a pedobarographic gait analysis and a fluoroscopic examination at a single follow-up appointment during routine postoperative control visits. Via fluoroscopy, the remaining sagittal range of motion (ROM) of the adjacent joints at the arthrodesed ankle was measured. Gait analysis was performed for both the operated and the nonoperated foot for intraindividual control. The parameters surveyed included peak force, peak pressure, and contact time during the roll-over process to gain information about the load distribution of different zones of the foot.

### Pedobarographic Gait Analysis

Pedobarographic gait analysis was performed using the emed-c50 system (novel GmbH, Munich, Germany), a pedobarographic measurement platform with 4 capacitive sensors/cm^2^. The sensors measure vertical ground reaction forces and pressure distribution of the bare foot under dynamic conditions. The platform was situated in the middle of an 8 m long, even walkway. The area of the foot was divided automatically by the software into 10 zones: heel, midfoot, metatarsal bones 1 to 5 (each), toe 1, toe 2, and toes 3 to 5. Measurements included peak force (N), contact area (cm^2^), peak pressures (kPa), and contact time in percentage of the roll-over process (% ROP). Peak forces were normalized by the weight of the respective test person and are given as a percentage of body weight (% BW). Values were provided for each zone and for the entire foot. Measured data were transmitted to a personal computer, where data were stored and processed using the emed report professional software (version 23.3.24).

Patients were asked to walk barefoot at a normal, self-selected speed and step length over the walkway. To obtain a normal gait pattern, each patient completed several test runs to determine the individual starting point. The measurement was considered complete if the rolling process of the foot under investigation was recorded in full by the measuring platform. Five complete sets of measurement data were taken from the operated as well as from the nonoperated foot for further evaluation.

### Fluoroscopic Analysis

**Figure 1.**
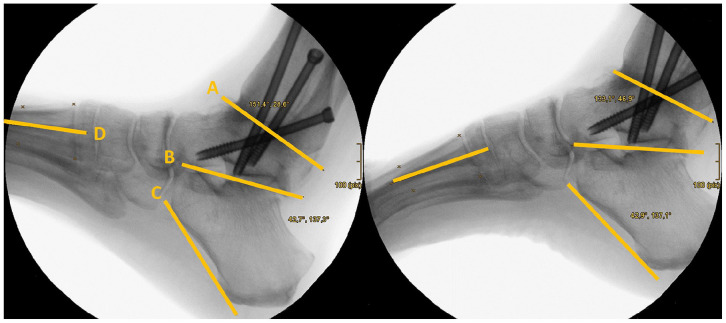
Lateral radiographs of the arthrodesed ankle in (a) maximum dorsiflexion and (b) maximum plantarflexion. Shown are the auxiliary lines marking bony landmarks of (A) tibia, (B) talus, (C) calcaneus, and (D) metatarsal 1, used for determining the range of motion (ROM) of the subtalar and medial tarsal joints. (complete ROM: difference of angles between A or B and D, subtalar ROM: difference of angles between A or B and C).

As part of the postoperative radiologic controls, strictly lateral radiographs of the foot and ankle were taken in maximum dorsiflexion and maximum plantarflexion. The movement was guided by the examiner. To determine the range of motion of the subtalar and adjacent midfoot joints (talonavicular, naviculocuneiform, and cuneiform-metatarsal-1 joint) in the sagittal plane, the angle between the tibia and the longitudinal axis of the first metatarsal was measured. Because the tibial axis was not fully depicted on the image section, reliable bony landmarks were selected instead to draw a radiographic line on the distal tibia or the fused talus (see Figure 1). The first measurement was performed in maximum dorsiflexion and the second measurement in maximum plantarflexion. The difference between the 2 values gave the total sagittal range of motion. In order to further differentiate the compensation movements, the range of motion of the subtalar joint in dorsiflexion-plantarflexion was also determined. For this purpose, the angle between an auxiliary line on the lower edge of the calcaneus and the tibial line was measured (see Figure 1). The difference between this angle and the total range of motion was assumed to be the range of motion of the medial tarsal column. The axes and auxiliary lines used for measurement were drawn manually with the support of measurement tools in the radiographic software. All measurements were taken by the same examiner according to the defined criteria. The measurement technique has been described previously, particularly reporting a good inter- and intraobserver reliability.^19^

### Data Processing and Statistics

For pedobarography, the measurement results of the 5 trials per foot were automatically averaged by the software and presented in tabular form. All measured variables were used in a paired comparison for each patient, so that the nonoperated foot was used as an individual reference to the operated foot. Therefore, a paired *t* test was used for statistically testing if data were normally distributed; otherwise, a Wilcoxon signed-rank test was used. To test for normal distribution, a Shapiro-Wilk test was used. For bivariate correlation analysis, a scatter diagram was performed, using then the Pearson correlation coefficient *r* for normally distributed data or the Spearman correlation coefficient *ρ* for not normally distributed data. The pedobarographic and fluoroscopic evaluations were performed independently of each other in a masked fashion. Because of its retrospective design, no sample size calculation and power analysis was performed. *P* was set at .05 for all testing, using SPSS, version 28.0.1.1 (IBM Corp, Armonk, NY).

## Results

### Study Population

Thirty ankles from 29 patients (21 men, 8 women) met the inclusion criteria. The mean age was 60.8 (range, 33-79) years, the mean follow-up was 14 (SD, 7.3, 95% CI of mean 11.4-16.9) months. For pedobarographic analysis, 3 data sets were incomplete and thus had to be excluded. One patient with AAA of both ankles had also to be excluded from pedobarography, lacking a nonoperated foot for side-by-side comparison, leaving 25 data sets for analysis. Fluoroscopic images were available for 27 operated ankles.

### Pedobarographic Gait Analysis

#### Peak forces

Peak forces of the entire foot were significantly lower on the operated foot in comparison to the nonoperated foot. This decrease was most pronounced in the area of the great toe (−4.0% BW), followed by the first metatarsal (−3.1% BW) and the lesser toes (−1.2% and −1.8% BW; all *P* < .05). In contrast, peak forces under metatarsals 2, 4, and 5 increased slightly, but not significantly, whereas peak forces under the midfoot, heel, and metatarsal 3 remained almost unchanged compared with the nonoperated side. Results are shown in Table 1.

**Table 1.** Peak Forces (% Body Weight) From Pedobarographic Measurements for All Zones of the Foot in Side-by-Side Comparison (Operated vs Nonoperated Foot).^a^

	Peak Force (% BW)				
	Operated Foot	Nonoperated Foot	Mean Difference	95% CI of Mean Difference	*P*
Entire foot	110.1 (5.6)	112.6 (6.9)	−2.5	−4.2 to −0.8	.007^b,*^
Heel	62.3 (7.3)	60.6 (11.8)	+0.5	−4.0 to 5.0	.816^b^
Midfoot	23.7 (10.7)	23.4 (11.1)	+0.5	−3.1 to 4.1	.774^b^
MT 1	18.7 (6.1)	20.1 (6.3)	−3.1	−5.8 to −0.3	.032^b,*^
MT 2	25.1 (7.5)	25.6 (6.5)	+1.1	−1.4 to 3.6	.377^b^
MT 3	24.8 (5.5)	24.6 (5.6)	+0.3	−1.4 to 2.1	.699^b^
MT 4	17.6 (4.0)	15.1 (3.9)	+1.7	−0.1 to 3.5	.060^b^
MT 5	10.1 (4.2)	7.9 (3.9)	+1.4	−0.3 to 3.0	.094^c^
Greater toe	11.1 (7.0)	14.7 (8.9)	−4.0	−6.8 to −1.2	.007^b,*^
Toe 2	2.8 (2.3)	4.3 (2.0)	−1.2	−2.3 to −0.1	.003^c,*^
Toes 3-5	3.1 (3.4)	4.8 (3.0)	−1.8	−2.9 to −0.7	.001^c,*^

Abbreviation: MT, metatarsal.

a Data are presented as mean (SD). Mean difference: operated vs nonoperated foot.

b Paired *t* test.

c Wilcoxon signed-rank-test.

* *P* < .05 vs control.

#### Peak pressure

**Figure 2.**
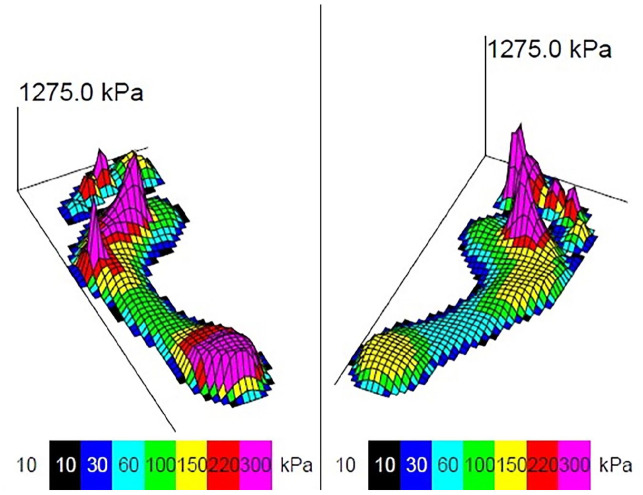
Example pressure profile of a patient with arthrodesis of the left ankle (shown left in the image). A shift of peak pressure to the lateral forefoot in the area of metatarsal 5 and a decrease of peak pressure under the great toe can be seen.

In side comparison, peak pressure increased significantly under metatarsal 5 (+85.9 kPa), whereas a significant decrease could be detected for the lesser toes (−51.3 and −60.3 kPa, all *P* < .05). Peak pressure also decreased under metatarsal 1 and the great toe, but not significantly. Interestingly, there was no significant difference in peak pressure for midfoot, heel, and metatarsals 2 and 3 (Table 2 and Figure 2).

**Table 2.** Peak Pressure Measured in Kilopascals From Pedobarographic Measurements for All Zones of the Foot in Side-by-Side Comparison (Operated vs Nonoperated Foot).^a^

	Peak Pressure (kPa)				
	Operated Foot	Nonoperated Foot	Mean Difference	95% CI of Mean Difference	*P*
Entire foot	753.5 (263.3)	772.1 (233.3)	−18.6	−138.2 to 101.0	.750^b^
Heel	332.8 (99.6)	326.9 (78.8)	+5.9	−24.1 to 35.9	.687^b^
Midfoot	174.2 (111.1)	163.8 (52.5)	+10.4	−40.2 to 61.1	.761^c^
MT 1	305.1 (188.7)	367.1 (237.3)	−61.9	−163.1 to 39.2	.128^c^
MT 2	542.1 (267.4)	551.9 (275.0)	−9.8	−115.9 to 96.3	.927^c^
MT 3	451.4 (141.1)	487.8 (180.1)	−36.4	−115.2 to 42.3	.503^c^
MT 4	309.3 (87.5)	298.8 (91.8)	+10.5	−41.0 to 61.9	.315^c^
MT 5	431.1 (309.3)	345.2 (255.9)	+85.9	−6.3 to 178.1	.045^c,*^
Great toe	369.1 (264.4)	475.5 (263.4)	−105.4	−223.9 to 13.1	.055^c^
Toe 2	170.8 (120.1)	222.1 (101.2)	−51.3	−98.5 to −4.2	.034^b,*^
Toes 3-5	134.6 (135.3)	194.9 (111.4)	−60.3	−116.8 to –3.8	.006^c,*^

Abbreviation: MT, metatarsal.

a Data are presented as mean (SD). Mean difference: operated vs nonoperated foot.

b Paired *t* test.

c Wilcoxon signed-rank-test.

* *P* < .05 vs control.

#### Contact time

The contact time as a proportion of the entire roll over process was significantly shorter for the entire forefoot (metatarsals 2-5 and lesser toes 2-5), except the first ray (Table 3).

**Table 3.** Contact Times (% ROP) From Pedobarographic Measurements for All Zones of the Foot in Side-by-Side Comparison (Operated vs Nonoperated Foot).^a^

	Contact Time (% ROP)				
	Operated Foot	Nonoperated Foot	Mean Difference	95% CI of Mean Difference	*P*
Entire foot	100	100			
Heel	59.6 (11.7)	59.3 (8.2)	+0.3	−4.1 to 4.7	.882^b^
Midfoot	64.8 (9.2)	66.8 (6.8)	−2.0	−6.0 to 2.0	.312^b^
MT 1	76.9 (5.9)	78.2 (5.4)	−1.2	−3.0 to 0.5	.162^b^
MT 2	79.8 (5.7)	81.6 (5.6)	−1.8	−3.1 to −0.5	.009^b,*^
MT 3	82.6 (4.9)	84.3 (4.5)	−1.6	−2.9 to −0.4	.011^b,*^
MT 4	81.1 (4.1)	83.1 (3.8)	−2.0	−3.6 to −0.5	.013^b,*^
MT 5	75.0 (4.7)	77.8 (3.8)	−2.8	−4.9 to −0.7	.011^b,*^
Great toe	61.1 (15.0)	67.3 (11.2)	−6.2	−14.1 to 1.7	.086^c^
Toe 2	49.0 (19.1)	58.8 (13.1)	−9.8	−16.3 to −3.3	.006^c,*^
Toes 3-5	49.3 (24.8)	63.4 (16.4)	−15.1	−23.4 to −6.7	<.001^c,*^

Abbreviation: MT, metatarsal; ROP, roll-over process.

a Data are presented as mean (SD). Mean difference: operated vs nonoperated foot.

b Paired *t* test.

c Wilcoxon signed-rank-test.

* *P* < .05 vs control.

Compensation Movements of the Subtalar and Midfoot Joints

**Figure 3.**
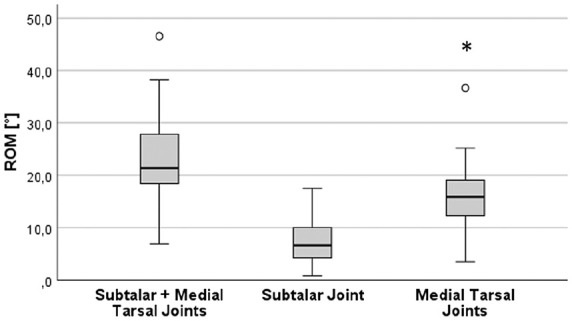
Range of motion of the subtalar joint, medial tarsal joint, and complete ROM. *The ROM of the medial tarsal column is significantly higher (15.9 degrees, SD 6.9) than the mean ROM of the subtalar joint (7.7 degrees, SD 4.4; paired *t* test, *P < .001).

The mean sagittal range of motion (ROM) of the adjacent joints was 23.5 (SD 8.5) degrees. The ROM of the medial tarsal column was significantly higher (15.9; SD 6.9 degrees) than the mean ROM of the subtalar joint (7.7; SD 4.4 degrees; paired *t* test, *P* < .001) (Figure 3). Neither age nor sex had an influence on the ROM of any of the analyzed joints (paired *t* test, *P* = .618 for sex; Pearson *r* = 0.102, *P* = .612 for age). The ROM, especially of the subtalar joint, increased with increasing time from the operation, but this trend did not reach the significance level (Spearman *ρ* = 0.326, *P* = .097).

### Relationship Between Compensation Movements and Gait Cycle

**Figure 4.**
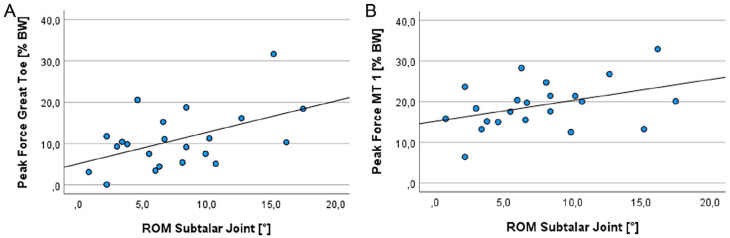
Correlation analyses between the ROM of the subtalar joint and the peak forces of the (A) great toe and (B) metatarsal (MT) 1. Panel A shows a significant positive linear relationship between the ROM of the subtalar joint and the peak forces exerted by the great toe (Pearson *r* = 0.497, *P* = .019). Panel B indicates a trend between the ROM of the subtalar joint and the peak forces exerted by metatarsal 1 (Pearson *r* = 0.406, *P* = .061).

**Figure 5.**
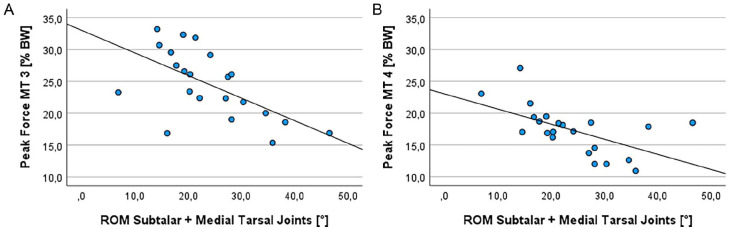
Correlation analysis between the ROM of the adjacent joints and the peak forces of (A) metatarsal (MT) 3 and (B) metatarsal 4. The peak forces decreased significantly (Pearson *r* = −0.609 and −0.574, *P* = .003 and .005).

The correlation analyses showed a significant positive linear relationship between the ROM of the subtalar joint and the peak forces of the great toe (Figure 4A; Pearson *r* = 0.497, *P* = .019). This relationship is also shown as a nonsignificant trend for metatarsal 1 (Figure 4B; Pearson *r* = 0.406, *P* = .061). In contrast, peak forces decreased significantly for metatarsals 3 and 4 by increasing the ROM of all adjacent joints (Figure 5; Pearson *r* = −0.609 and −0.574, *P* = .003 and .005). This correlation was also confirmed with regard to contact times, as there was also a significant, positive linear correlation between the overall mobility of the neighboring joints and the contact time of the greater toe (Spearman *ρ* = 0.546, *P* = .009).

## Discussion

In the present study, we analyzed the residual mobility of the midfoot joints after AAA, as well as the load distribution and alterations during walking to test whether mobility influences load and gait pattern.

Various studies have analyzed the gait pattern of the arthritic as well as the arthrodesed ankle joint, especially in regard of spatiotemporal variables. Because of joint stiffness and pain, patients with osteoarthritis of the ankle spend less time on the affected limb during stance, slowing down the walking speed, as well as reducing cadence and stride length.^1^ Those gait abnormities are improved by ankle arthrodesis.^4,6,8^ The walking speed fastens, but still differs from normal gait. Cadence and stride length still remain reduced.^1,14,22,28^ In regard to joint motion, most studies report a decreased range of motion of the “ankle” after arthrodesis, but do not specify the analyzed joints.^3,22,23^ This may be due to the selected gait analysis methods, mainly 3-dimensional video-assisted systems, which make it difficult to differentiate between the joints. As a reduced ROM after arthrodesis of a joint is not surprising, studies with a more detailed analysis of the ROM of the adjacent joints present contrary results. Sealey et al,^19^ analyzing the sagittal ROM of the subtalar and medial tarsal joints via lateral stress radiographs pre- and postarthrodesis, report a significant increase of 10.8% after arthrodesis for the combined joint motion. Even when considered separately, both the subtalar joint and the medial tarsal column showed a significant increase in movement (from 5.2 to 9.3 degrees and from 14.3 to 16.4 degrees, respectively). Also comparing pre- and postoperative joint mobility, Brodsky et al report a constant range of sagittal motion (from tibia to forefoot) after arthrodesis, despite the loss of tibiotalar mobility. Thus, a greater mobility of the subsequent joints can be assumed.^4^ In the present study, the mean sagittal ROM of the subtalar and tarsal joints was 23.5 degrees, with 7.7 degrees resulting from the subtalar and 15.9 degrees from the tarsal joints. These results are consistent with the available literature, all presenting a residual ROM of this segment between 22 and 24 degrees, with also comparable proportion of the subsegments.^2,13,19^ In a large series of arthritic ankles, Valderrabano et al^24^ reported a sagittal ROM of the foot and ankle of 22 degrees. Thus, all the above-mentioned studies measuring the ROM postarthrodesis achieve greater joint mobility than the arthritic, but nonfused, foot. A more recent study, using finite element analysis for simulation of various biomechanical parameters after ankle arthrodesis, also came to the conclusion that the sagittal ROM of the forefoot after arthrodesis is larger than in the intact foot.^25^

Our study showed a trend of increasing subtalar mobility with increasing time from the operation. In the work from Sealey et al,^19^ an initial decrease in the mobility of the adjacent joints was observed 6 months after arthrodesis, which increased significantly in the further course up to the final follow-up after 33 months. In our study, the follow-up time varied considerably, which may be one reason for this nonsignificant result. A longer follow-up might have reinforced this trend above the significance level. Although dorsiflexion/plantarflexion is not the main direction of movement of the subtalar joint, it seems that particularly the mobility of this joint increases after arthrodesis of the tibiotalar joint.

This is possibly an expression of an adaptation process to compensate for the loss of mobility of the tibiotalar joint and therefore also limited inversion and eversion of the subtalar joint and a possible cause of the subsequent subtalar arthrosis that develops over time. This also illustrates the importance of considering the mobility of the adjacent joints in the context of postoperative rehabilitation and physiotherapy.

In contrast to an increasing mobility of the subtalar joint, peak pressure analysis in the present study could not reveal a significant increase under the midfoot and the hindfoot. A significant increased peak pressure could only be detected under metatarsal 5, whereas the load of the toes and the medial metatarsals decreased. These results are in concordance with the results of Schuh et al^18^ and demonstrate that peak pressure distribution is altered only to a small extent by arthrodesis. A more recent finite element analysis by Wang et al,^25^ calculating peak pressure and joint contact pressure, estimated higher and anteriorly shifted peak pressures. Although this could not be confirmed in our analysis, the authors also stated a lateral displacement of peak pressure during toe-off.

A shift in the center of maximal load has also been described regarding ground reaction forces.^1,2^ Beyaert et al^2^ found out that after ankle arthrodesis, ground reaction forces shifted forward during midstance in comparison to controls, but with the peak posterior to the metatarsal heads during toe-off. These findings are in concordance with the results of our study. Peak forces decreased significantly under the great toe, the lesser toes, and the first metatarsal, indicating that after AAA, the toe-off is less powerful. Peak forces increased slightly under the central and lateral metatarsals. Generally, peak forces of the entire foot were reduced in comparison to the contralateral foot, which is in concordance with the literature and may be due to general muscle weakness and protection of the operated limb.^1,10,18^

To our knowledge, we are the first to analyze the influence of the midfoot mobility on gait pattern and load distribution. We could show a positive correlation between the ROM of the adjacent joints and peak forces, as well as contact time of the great toe. In contrast, there was a negative correlation between the ROM and peak forces of the lateral metatarsals. The greater the ROM of the adjacent joints, the more is the gait pattern normalized.

Our study has several limitations. With the aid of pedobarographic gait analysis, it was not possible to gain any insights into spatiotemporal gait changes, with the exception of contact time, although these were investigated in detail in earlier studies. However, it cannot be ruled out that the walking speed, for example, also has an influence on the parameters investigated. Because we used the nonoperated foot for control, we do not know about existing osteoarthritis or other radiologic alterations in those feet. The maximum range of motion of the adjacent joint was determined under lateral radiographs, thus only analyzing sagittal ROM. As no body load was applied, we do not know whether the full ROM we measured is used during walking. In addition, the position of the joint partners changes under load, which could alter the measurements. Nevertheless, all studies with detailed analysis of subtalar and tarsal joint mobility after arthrodesis report almost identical results, regardless of whether the measurement was taken on the loaded or unloaded foot.^2,13,19^ Thus, the influence may be minor. Furthermore, as the follow-up time is relatively short, a further change in the ROM over time with a prolonged follow-up is possible.

## Conclusion

After AAA, changes in gait can be seen with a reduced load of the operated foot, and especially a less powerful toe-off of the first ray. This deficit is compensated by a higher load on the lateral forefoot, but no significant increase of midfoot and hindfoot load. With higher ROM of the subtalar and tarsal joints, gait parameters normalize.

## Supplemental Material

sj-pdf-1-fao-10.1177_24730114251338848 – Supplemental material for Compensatory Movements of the Midfoot Joints Influence Gait Pattern After Arthroscopic Ankle Arthrodesis Supplemental material, sj-pdf-1-fao-10.1177_24730114251338848 for Compensatory Movements of the Midfoot Joints Influence Gait Pattern After Arthroscopic Ankle Arthrodesis by Annette Eidmann, Katharina Kraftborn, Matthias G. Walcher, Lukas Fraißler, Maximilian Rudert and Ioannis Stratos in Foot & Ankle Orthopaedics
